# A Cross-Sectional Study on Pornography Use and Sex Differences Among High School ‎Students

**DOI:** 10.7759/cureus.74482

**Published:** 2024-11-26

**Authors:** Ali Jotiar Mahmood, Hajar Hassan Abdulqadir, Rojeen Chalabi Khalid, Vindad Hashim Dirbas, Zana Sherwan Ahmed, Kareen Yarwant Naisan, Duaa Farhad Hasan

**Affiliations:** 1 College of Medicine, University of Zakho, Zakho, IRQ; 2 College of Medicine, University of Duhok, Duhok, IRQ

**Keywords:** associated factors, high schools, institutions, pornography, student's perspective

## Abstract

Background and aim: Pornography consumption is on the rise globally due to the widespread availability of the internet and unrestricted access to numerous pornographic websites. These sites pose various complications for consumers, and there is a noticeable lack of research on this issue in our country. Therefore, it is thought necessary to conduct this study. This study aims to measure pornography use among high school students, sex differences in pornography viewing, and the behavior of pornography viewing in the Kurdistan Region, Iraq.

Materials and methods: This cross-sectional study was conducted at Zakho Independent Administration, Kurdistan Region, Iraq, among students of five high schools and two institutions. A paper questionnaire was handed out to the participants between April 1 and July 1, 2022. Descriptive statistics and inferential analyses, including chi-square tests, were utilized to examine sex differences and explore associations between the variables.

Results: A total of 921 students were enrolled in this study, with an average age of 16.78 ± 1.26 years. More than half of the participants were male, 505 (54.83%); the ones who viewed pornography alone were 452 (49.08%), and 642 (69.71%) disagreed with watching porn. The first time watching pornography was more common at the age of 15-20, with over one-fourth of participants, 254 (27.58%), falling into this age group. There were statistically significant differences (p < 0.001) in the attitude and practice of men compared with women throughout all tested variables. Out of the total number of participants, 467 (50.71%) viewed pornography at least once in their lifetime; among those, 304 (65.1%) were male participants, 163 (34.9%) of them were female participants, and 852 (92.51%) participants agreed to restrict pornography sites.

Conclusions: The prevalence of pornography among high school students is relatively high. Male sex is an associated factor for higher pornography viewing and should be considered when designing public health interventions in a related context. Increasing education about this topic in high school settings is crucial.

## Introduction

There is no specific definition for pornography, but it could be defined as printed or visual material containing the explicit description or display of sexual organs or activity that has the primary intention of sexually arousing the consumer [1]. With the increased availability of the internet, pornography has become easily accessible and inexpensive for people of all ages and sexes, as it no longer requires printing or downloading [2]. Internet pornography consumption may serve as a defense mechanism against excessive stress, helping individuals cope with stressful events, regulate their mood, and reduce feelings of depression and anxiety [3]. Pornography consumption is regarded as a problem because it is associated with many issues like sexual dysfunction, especially at an early age, and also associated with mental health problems through reducing memory [4,5]. There is a relationship between sexual violence and pornography usage [6]. One of the surveys that has been done among 47 high school students showed that frequent use of pornography is associated with more attraction to different sexual interests, such as sex as a group and anal sex [7]. Exposure to pornography has been associated with adolescent dating violence and sexual aggression [8].

A cross-sectional study conducted in Arabic countries revealed that men consume pornography more than women [9]. Another study conducted in Poland reported that 202 respondents (76.8%) admitted to using online pornography, of which 122 (93.1%) were men and 80 (60.6%) were women [10]. Another research has been done in Australia: 815 (87%) of the participants have viewed pornography, 257 (100%) of the male participants viewed pornography at least one time in their life, while female participants, 558 (82%), ever viewed pornography [11]. A survey done in Bangladesh showed that out of 313 students, 221 students (70.6%) consumed pornography at least once within their entire lives, among whom 188 (87.4%) were male participants, while only 33 (35.5%) were female participants [12]. A large survey conducted in Australia has reported that 8,369 male participants (84%) ever viewed pornography, while 5,471 female participants (54%) ever viewed it [13]. The rate of rape and violence in the world also increases among those consuming pornography by about 30% [14]. The rate of sexually transmitted diseases and hepatitis virus in the world, including Iraq, also increases among different age groups and sexes [15-19]. Many studies have been conducted in Kurdistan, Iraq, about sexually transmitted diseases, including infection-causing problems in the genital tract [20-22], but there is no study about pornography. Despite the increase in pornography consumption during the last decade, pornography viewing differs by sex and age group [23].

Due to the absence of a large-scale study in the Kurdistan Region, Iraq, the present study aimed to investigate pornography use among high school students and measure sex differences with pornography viewing and the behavior of pornography viewing.

## Materials and methods

Study design and participants

**Figure 1:**
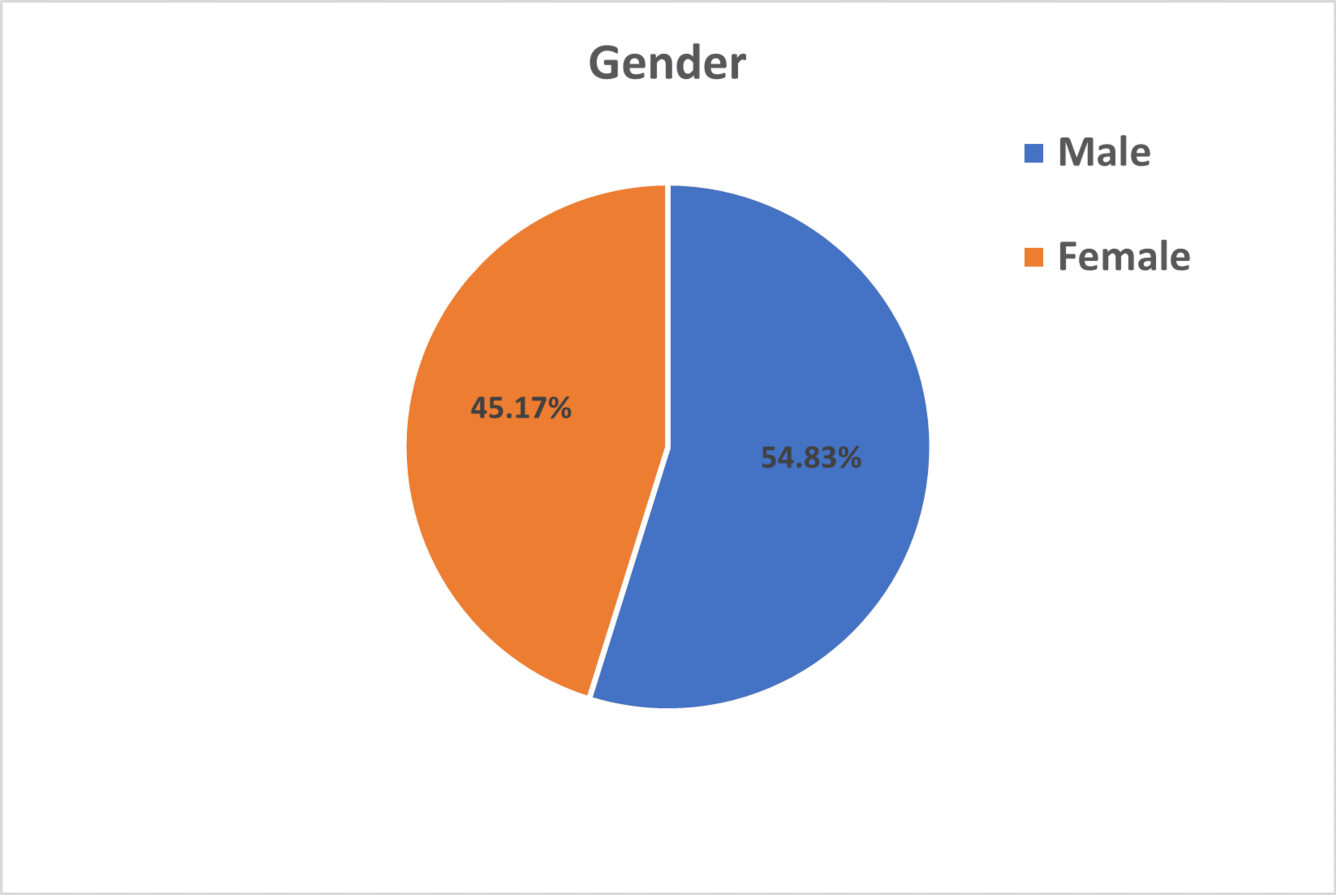
Sex differences in the study participants

This cross-sectional study was conducted randomly from five high school and two institution students in Zakho Independent Administration Kurdistan Region, Iraq. The participants were selected randomly, and a paper questionnaire was administered between April and July 2022. The total number of participants has been estimated based on the assumption that there are approximately 20,000 students enrolled in high schools. An online calculator, http://www.raosoft.com/samplesize.html, was used to determine this estimate, which has a confidence interval of 99% and a margin of error of 5%, resulting in a sample size of 643 participants. However, we have enrolled 921 to decrease the chance of bias, with a mean age of 16.78 years and a standard deviation (SD) of ±1.26. Most participants were male, 505 (54.83%) (Figure 1).

Study survey

The questionnaire was taken from other surveys conducted in Arab countries [8]. The questionaries were printed to be distributed among the students in the different schools. Then, we visited each class, and students filled out the questionaries confidentially from the other students by keeping a distance between them. The cover for the questionnaire was shuffled in front of them when they returned.

The first part of the questionnaire is about the demographic characteristics of participants, including sex, address, and age. The second part contained information regarding their frequency of use, time consumption of pornography, and first exposure to pornography. The last part of the questionnaire contained questions about their views on pornography, such as whether they believe it should be forbidden and their opinions on watching it. Unfinished surveys and students who did not agree to participate in this study were excluded.

Ethical consideration

The protocol, design, and procedure of the study were approved by the Scientific and Ethics Committee of the College of Medicine, University of Zakho, Duhok Province, Kurdistan Region, Iraq (Ethic Committee reference number: 4/154/NW). Written informed consent was obtained from all participants, and the ethics committee waived parental consent since we had not done any procedure.

Statistical analysis

All data were analyzed using GraphPad Prism version 8.0 (GraphPad Software, Inc., San Diego, CA). Standard descriptive statistics were calculated for each question as frequencies and percentages, and continuous variables were calculated as mean and SD. The chi-square test was used to estimate the sex differences with each question individually. A p value of <0.05 was considered statistically significant.

## Results

The total number of participants enrolled in this study was 921, with a mean age of 16.78 years and an SD of ±1.26. Of the total number of participants, 505 (54.83%) were male participants, and 416 (45.17%) were female participants (Figure 1). The agreement regarding the viewing of pornography indicated that 642 (69.71%) participants strongly disapproved of watching pornography, with 328 (51.09%) being female participants (Table 1). However, 308 (33.44%) participants believed that more than 80% of men view pornography, with the majority being male participants, totaling 204 (66.23%) (Table 1). Of the total participants, 254 (27.58%) started to view pornography between the ages of 15 and 20 years; 266 (28.88%) were exposed to pornography accidentally, among whom 136 (51.13%) were male participants and 130 (48.87%) were female participants; 99 (10.75%) participants were exposed to pornography through intentional searching, where 84 (84.85%) were male participants and 15 (15.15%) were female participants. Of the total participants, 164 (17.81%) reported using pornography less than one time per month in the last 12 months. In the last month, 140 (15.2%) participants have consumed pornography between one and five times, and in the last seven days, 75 (8.14%) participants consumed pornography between one and five times.

**Table 1:** Attitude and practice among sex differences in watching pornography p value <0.05 is considered significant p value is determined by chi-square test (Fisher's exact test)

Variables		Total, N (%)	Sex, N (%)		p value	Chi-square
			Male	Female		
Do you agree on watching porn?	Strongly agree	17 (1.85)	12 (70.59)	5 (29.41)	0.001	32.63
	Agree	14 (1.52)	12 (85.71)	2 (14.29)		
	Don’t know	74 (8.03)	53 (71.62)	21 (28.38)		
	Disagree	174 (18.89)	114 (65.52)	60 (34.48)		
	Strongly disagree	642 (69.71)	314 (48.91)	328 (51.09)		
In your opinion, what is the percentage of men watching porn?	<20%	6 (0.65)	2 (33.33)	4 (66.67)	0.001	37
	<50%	43 (4.67)	22 (51.16)	21 (48.84)		
	>50%	197 (21.39)	113 (57.360)	84 (42.64)		
	>80%	308 (33.44)	204 (66.23)	104 (33.77)		
	100%	69 (7.49)	37 (53.62)	32 (46.38)		
	I don’t know	298 (32.36)	126 (42.28)	172 (57.72)		
In your opinion, what is the percentage of women watching porn?	<20%	19 (2.06)	10 (52.63)	9 (47.37)	0.001	30.15
	<50%	218 (23.67)	114 (52.29)	104 (47.71)		
	>50%	183 (19.87)	100 (54.64)	83 (45.36)		
	>80%	86 (9.34)	67 (77.91)	19 (22.09)		
	100%	28 (3.04)	22 (78.57)	6 (21.43)		
	I don’t know	387 (42.02)	191 (49.35)	196 (50.65)		
Your age at your first time of watching porn	<10	8 (0.87)	7 (87.5)	1 (12.5)	0.001	44.29
	10-15	204 (22.15)	139 (68.14)	65 (31.86)		
	15-20	254 (27.58)	157 (61.81)	97 (38.19)		
	20-25	1 (0.11)	1 (100)	0 (0)		
	Never	454 (49.29)	201 (44.27)	253 (55.73)		
How were you exposed to porn for the first time	Intentional searching	99 (10.75)	84 (84.85)	15 (15.15)	0.001	67.35
	A friend	45 (4.89)	40 (88.89)	5 (11.11)		
	A family member	6 (0.65)	3 (50)	3 (50)		
	Accidently	266 (28.88)	136 (51.13)	130 (48.87)		
	Others	505 (54.83)	246 (47.92)	263 (52.08)		
In the last 12 months, how many times did you watch porn?	Daily	24 (2.61)	24 (100)	0 (0)	0.001	138.14
	Weekly	75 (8.14)	71 (94.67)	4 (5.33)		
	Monthly	52 (5.65)	43 (82.69)	9 (17.31)		
	<1 time per month	164 (17.81)	113 (68.9)	51 (31.1)		
	Never	606 (65.8)	254 (41.91)	352 (58.09)		
In the last months, how many times did you watch porn?	>10 times	36 (3.91)	35 (97.22)	1 (2.78)	0.001	148.54
	6-10	29 (3.15)	28 (96.55)	1 (3.45)		
	1-5	140 (15.2)	119 (85)	21 (15)		
	None	716 (77.74)	323 (45.11)	393 (54.89)		
In the last seven days, how many times did you watch porn?	>10 times	9 (0.98)	9 (100)	0 (0)	0.001	45.44
	6-10	16 (1.74)	15 (93.75)	1 (6.25)		
	1-5	75 (8.14)	62 (82.67)	13 (17.33)		
	None	821 (89.14)	419 (51.04)	402 (48.96)		
With whom did you watch porn?	None	448 (48.64)	200 (44.64)	248 (55.36)	0.001	44.54
	Alone	452 (49.08)	284 (62.83)	168 (37.17)		
	Friend(s)	9 (0.98)	8 (88.89)	1 (11.11)		
	Life partner	8 (0.87)	8 (100)	0 (0)		
	Other	4 (0.43)	4 (100)	0 (0)		
A visit to the psychiatrist in the last 12 months	Yes	28 (3.04)	16 (57.14)	12 (42.86)	0.84	0.06
	No	893 (96.96)	489 (54.76)	404 (45.24)		
Do you agree to close these sites?	Yes	852 (92.51)	462 (54.23)	390 (45.77)	0.21	1.69
	No	69 (7.49)	43 (62.32)	26 (37.68)		

**Figure 2:**
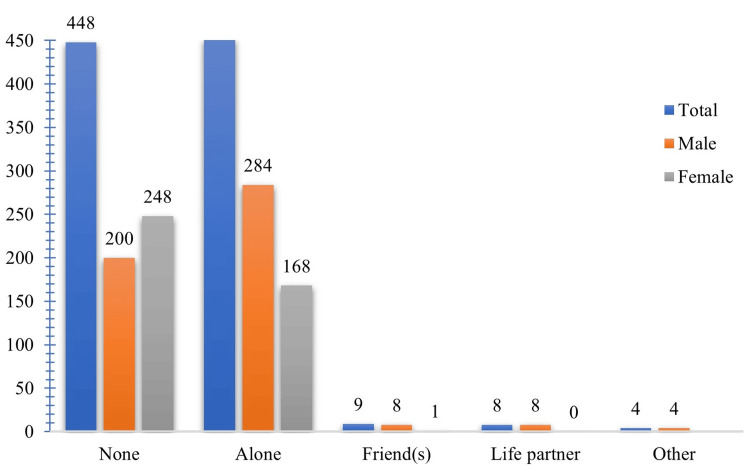
‎ Preference for viewing pornography based on companion type

Regarding those with whom they watched pornography, 452 (49.08%) participants reported watching it alone, among whom 284 (62.83%) were male participants and 168 (37.17%) were female participants (Figure 2). In the past 12 months, 16 (57.14%) male participants had visited the psychiatrist. Surprisingly, a total of 852 (92.51%) participants agreed to close the pornography sites, with 462 (54.23%) being male participants and 390 (45.77%) being female participants.

There were statistically significant differences (p < 0.001) in the attitude and practice of men compared with women throughout all tested variables (Table 1). This included their opinions on the act of watching porn, the percentage of men/women watching porn, the way of first exposure, the accompanying person, and the frequency as well.

## Discussion

According to our knowledge, this is the first survey that has been conducted in the Kurdistan Region of Iraq to know how many students in high school have been exposed to and are consumers of pornographic sites. In the current study, most participants were young, with a mean age of 16.78 years.

The experience of pornography in the last few years was as follows: 606 (65.8%) respondents reported that they had never viewed it, 164 (17.81%) participants viewed it less than once per month, 52 (5.65%) viewed it monthly, 75 (8.14%) viewed it weekly, and 24 (2.61%) viewed it daily. These data are lower than the other surveys that have been done, like the large multinational in Arabic countries [8] and another one in Australia [12]; this is because most of our participants are 15 years old, so they may not yet have been exposed to pornography. In our culture, watching pornography is considered shameful, and many participants did not trust that their identities would remain anonymous, leading to potential bias in their responses [24]. Among those who have consumed pornography, 254 (27.58%) participants were exposed for the first time between the ages of 15 and 20 years. Perhaps this age group is higher because of the neurobiological changes in adolescence age, which are an increase in dopamine levels, activation of penile erection, promotion of sexual drive with orgasmic quality, and increase of sexual hormone causing an increase in libido [25-27]. Furthermore, in our culture, sexual activity outside of marriage is forbidden [24]. Since all the participants are students and too young to marry, they may turn to pornography, which lacks restrictions, increasing the likelihood of viewing it. Of the total participants, 642 (69.71%) strongly disagree with watching pornography, which may be due to a cultural effect, the population having awareness about the effects of pornography, or a combination of both. Furthermore, 266 (28.8%) participants reported accidentally accessing pornographic sites, which increases the risk of addiction. This is because the body naturally has a reward system for behaviors like sex and eating. Since pornography is related to sexual behavior, the body responds with a similar reward system for it as well [28,29], so it will increase the number of addicted people and consumers [30]. A total of 505 participants, representing 54.83%, chose "other" in regard to how they were first exposed because of the limitation of the questionnaire. The ones that have never consumed pornography did not have an option. So the number of participants that have to choose "other" is 505 (54.83%), and the number of participants that have never viewed these sites is 454 (49.29), so it can be concluded that 51 (5.54%) participants use another way for watching pornography. Many who have consumed pornography are male participants, a trend that has also been reported in other surveys [8,10-12]. Among those who reported watching pornography through intentional searching, 84 (84.85%) were male participants and 15 (15.15%) were female participants. This difference may be attributed to the perception that it is considered more shameful for females to watch pornography compared to males.

The study was limited by a low number of participants and restricted to only high school students, not determining the factors increasing the viewing of pornography. The present study has a cross-sectional design; the long-term effects cannot be determined by observing the behavior and changes in the person's life and comparing them to nonpornographic viewers. The students were not under the impression that this subject would be discussed with them, which made the situation hard to explain to them and collect the data at the same time, and they did not have any idea regarding the confidentiality of the research. They did not trust this process and did not answer correctly. Also, the questionnaire has not been filled by us because of confidentiality, which also has increased the chance of inaccuracy. For the next research, it is better to include a wider age range; the schools should educate them that the process is highly private and no one will know their information.

## Conclusions

Pornography consumption varies significantly across age and sex, with men typically engaging more frequently than women and adolescents comprising a large proportion of consumers. This pattern of consumption may increase the risk of pornography addiction and erectile dysfunction. Increasing awareness of these risks, particularly within adolescent educational settings, is recommended. Additionally, educating parents on the use of internet family packages that restrict access to explicit content may be beneficial. It is also advised that internet service providers offer such packages by default, with unrestricted access available only to individuals over the age of 18.
